# Multiple Orthokeratinized Odontogenic Cysts: A Report of Two Cases and Review of the Literature

**DOI:** 10.1007/s12105-019-01042-0

**Published:** 2019-05-22

**Authors:** Hannah Crane, Philip Da Forno, Elena Kyriakidou, Paul M. Speight, Keith D. Hunter

**Affiliations:** 1 Academic Unit of Oral and Maxillofacial Medicine, Pathology and Surgery, School of Clinical Dentistry, 19 Claremont Crescent, Sheffield, UK; 2 University Hospitals of Leicester NHS Trust, Leicester, UK

**Keywords:** Orthokeratinized odontogenic cysts, Odontogenic cysts, Diagnosis, Pathology

## Abstract

Orthokeratinized odontogenic cysts (OOC) are developmental odontogenic cysts characterised by an orthokeratinized stratified squamous epithelial lining. They were originally believed to be part of the spectrum of Odontogenic Keratocyst, but are now considered to be a distinct entity. They are rare, making up approximately 1% of all odontogenic cysts and they usually occur singly. In this paper we present two new cases of multiple OOCs, and compare them to previous case reports of multiple lesions. The clinical and pathological features are discussed, along with possible diagnostic pitfalls.

## Introduction

Orthokeratinized Odontogenic Cyst (OOC) is a developmental odontogenic cyst characterised by a lining of orthokeratinized stratified squamous epithelium [1]. They were first identified by Wright in 1981 [2] and were originally thought to be part of the spectrum of Odontogenic Keratocyst (OKC) [3]. In 2005, the World Health Organisation (WHO) re-classified the OKC as Keratocystic Odontogenic Tumour (KCOT) due to its high recurrence rate and aggressive biology, but stated that the orthokeratinized form was not part of the spectrum of KCOT [4]. As odontogenic cysts were removed from the WHO 2005 classification, this left the OOC without a clearly accepted designation. In 2017, however, the WHO reintroduced cysts into the classification, and also determined that there was insufficient evidence to call the OKC a benign neoplasm and reverted back to OKC as the preferred terminology [5]. The OOC was included in the re-introduced section on odontogenic cysts, clearly separating it as a distinct entity from the OKC [1].

OOCs are distinct from OKCs as they recur less frequently, with a recurrence rate of less than 2% [1], in comparison to a recurrence rate as high as 28% for OKCs [5]. Although the change in terminology means the true prevalence of OOC is unknown, they appear to be rare. They have previously accounted for 10% of OKCs and therefore make up approximately 1% of all odontogenic cysts [1].

Histologically, OOC differs from OKC predominately by orthokeratinization and the presence of a granular cell layer, in comparison to the parakeratin observed in OKC [1]. In addition, in OOC the surface keratin layers do not show the corrugation seen in OKC and the basal cells are flat, rather than palisaded [1]. Whereas it is well established that about 5% of OKC may be associated with the Naevoid Basal Cell Carcinoma Syndrome (NBCCS) and may present with multiple lesions [5] no such association has been reported for OOC and multiple OOCs appear to be a rare occurrence.

In this paper we describe two cases of multiple and bilateral OOCs, followed by a review of the previous literature on multiple OOCs.

## Case 1

**Fig. 1:**
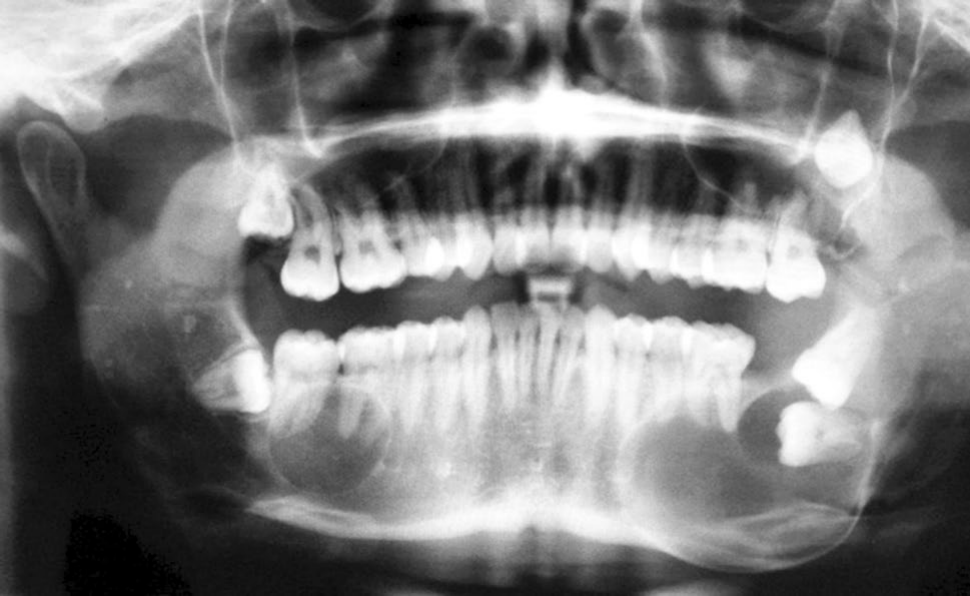
OPT from case 1 showing bilateral mandibular lesions and a cystic lesion associated with the displaced upper left 8 (UL8)

A 23-year-old male presented with three lesions, two in the mandible and one in the maxilla. An orthopantomogram (OPT) radiograph revealed three cystic lesions, the mandibular lesions were both well-defined and corticated (Fig. 1). In the right body of the mandible, a spherical radiolucency was noted overlying the roots of the lower right first and second molars. In the left body of the mandible, there was a larger radiolucent lesion with evidence of cortical expansion and displacement of the lower left second and third molars. A less well-defined radiolucency was seen in the left posterior maxilla. This lesion appeared to be multilocular and had inverted the third molar tooth and displaced it upwards into the posterior wall of the antrum.

**Fig. 2:**
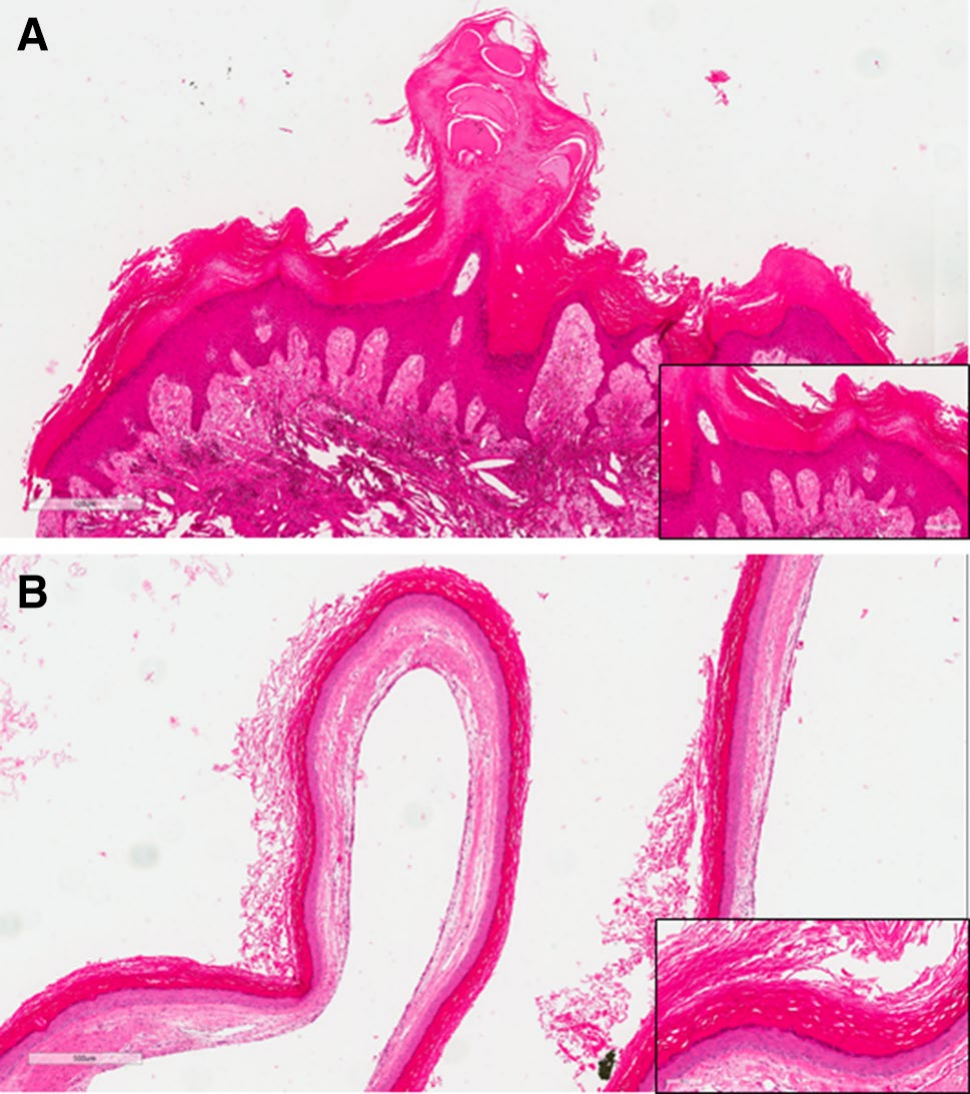
Histological appearance of the cysts in case 1. **a** Cyst from the left angle of mandible showing prominent orthokeratosis and a mild verrucous appearance, H&E stain, original magnification × 4. Insert bottom right, H&E stain, original magnification × 10. **b** Cyst from the right mandible showing prominent orthokeratosis, H&E stain, original magnification × 4. Insert bottom right, H&E stain, original magnification × 20

Histology of all three cysts revealed similar features (Fig. 2), showing cystic lesions with a fibrous connective tissue wall, lined by thin regular orthokeratinized stratified squamous epithelium. All three cysts showed evidence of chronic inflammation, with focal accumulations of foamy histiocytes and lymphocytes. Occasional cholesterol clefts and haemosiderin deposits were also noted and in these areas of inflammation, the lining was non-keratinised. In the cyst from the left mandible, there were also areas with very prominent orthokeratosis and a mild verrucous appearance (Fig. 2a).

The patient had no signs or symptoms to suggest NBCCS and did not meet the criteria for a diagnosis of NBCCS [6]. A diagnosis of multiple OOCs was made and conservative enucleation and curettage of the lesions was recommended. To date, there has been no evidence of recurrence at 48 months.

## Case 2

**Fig. 3:**
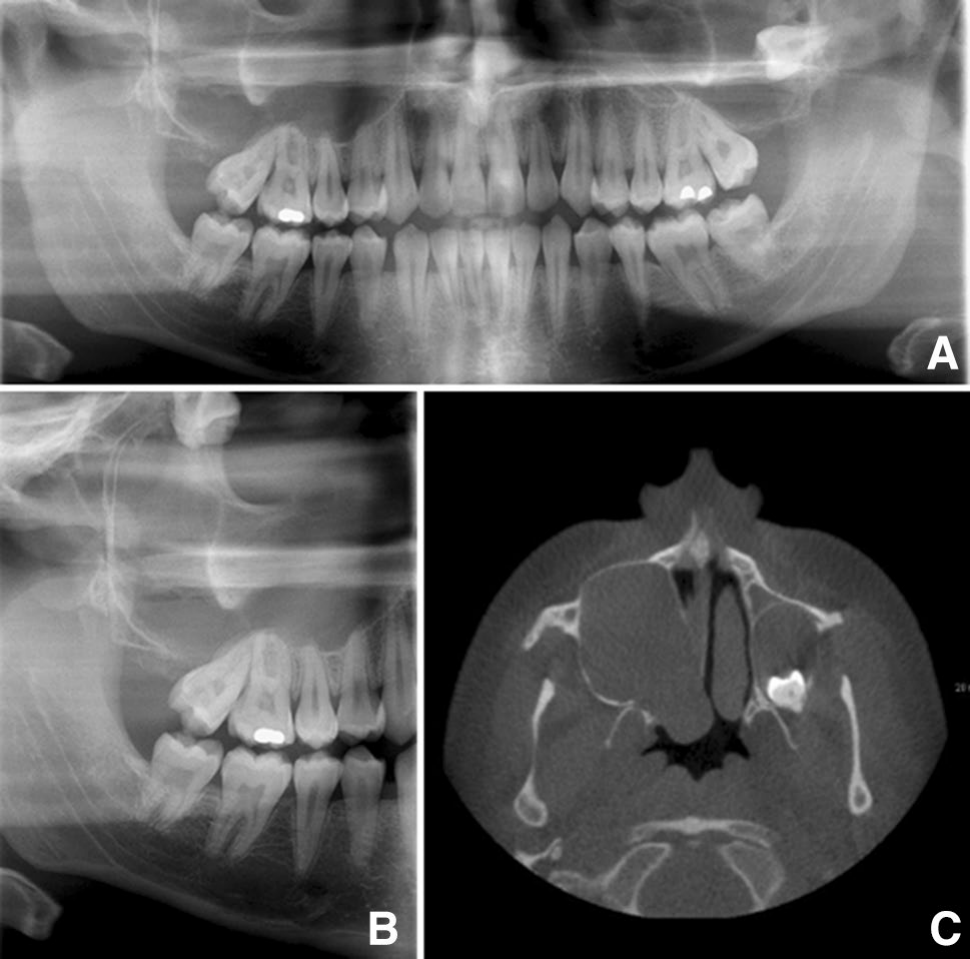
**a** Full OPT from case 2, showing the displaced upper left 8 (UL8) and associated radiolucency. Note that displaced upper right 8 (UR8) cannot be seen in this radiograph **b** Sectional OPT from case 2 showing displaced upper right 8 (UR8) with associated expansile radiolucency **c** CBCT from case 2 showing bilateral expansile lesions in the left and right maxilla

A 20-year male presented for extraction of a partially erupted and disto-angular positioned upper right second molar. On routine pre-operative radiographs, it was noted that both the left and right upper third molar teeth were impacted, displaced and associated with large, expansile radiolucencies (Fig. 3a, b). A Cone Beam CT (CBCT) revealed extensive expansion of both the right and left maxillary sinus by a soft tissue lesion (Fig. 3c), with the right lesion showing soft tissue extending up to the orbital floor. Both third molars had been displaced superiorly. The axial view (Fig. 3c) clearly shows the extensive medial and posterior expansion of the right maxillary sinus. The clinical differential diagnosis included OKC and dentigerous cyst.

The lesions were enucleated along with surgical removal of the maxillary impacted third molars.

**Fig. 4:**
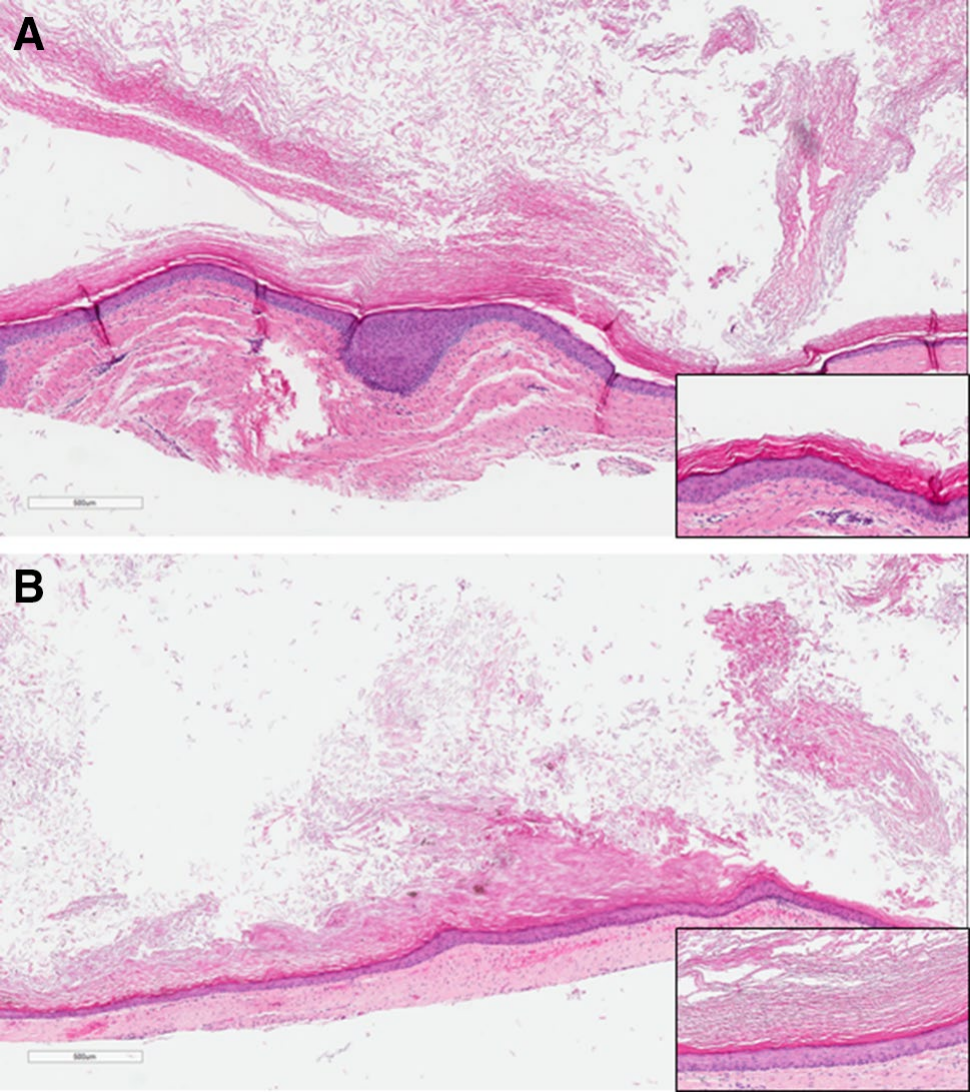
Histologically both lesions in case 2 showed a cyst lined by orthokeratinized stratified squamous epithelium **a** Cyst in left maxilla, H&E stain, original magnification × 4. Insert bottom right, H&E stain, original magnification × 20 **b** Cyst in right maxilla H&E stain, original magnification × 4. Insert bottom right, H&E stain, original magnification × 20

Histological examination of both the left and right specimens (Fig. 4) showed cystic lesions comprised of a dense fibrovascular connective tissue wall lined by orthokeratinized stratified squamous epithelium. For the most part the epithelium was thin and regular with no evidence of basal cell palisading or reversal of nuclear polarity. A prominent granular cell layer was present. Focal accumulations of chronic inflammatory cells were seen, along with deposits of haemosiderin and cholesterol cleft formation.

A diagnosis of bilateral OOCs was made. To our knowledge, there is no evidence of recurrence at 24 months.

## Discussion

These cases highlight the diagnostic difficulty of multiple OOCs, due to their clinical presentation being very similar to a number of other odontogenic lesions. In both cases OOC was not suggested in the clinical differential diagnosis, with the favoured diagnosis being OKC or dentigerous cysts.

Both cases occurred in males, which is in agreement with previous reports that show a male predominance [1, 7]. OOC are seen in the mandible two and a half times more frequently than in the maxilla [7], which makes the second case showing bilateral OOCs in the maxilla even more unusual. Both patients were in their third decade, which is in agreement with a systematic review which found that the largest proportion of OOC in western populations initially presented in the third decade [7]. In both patients, the cysts were incidental findings, whereas some cases of OOC in the literature, show they can be symptomatic at presentation, most commonly with swelling, pain or purulent discharge [8]. A systematic review in 2010, however, revealed that 48% of OOC presented as incidental findings, with a slightly lower percentage presenting with swelling (41%) [7]. As the majority of cases are incidental findings, careful review of all routine radiographs, especially OPTs, is indicated to assess for unexpected pathology.

A review of OOCs showed that 93% of cases were unilocular [7] and this is reflected in the cases presented here, with only the lesions in the left maxilla and mandible of case 1 (Fig. 1) showing a multilocular appearance. The cases presented here showed marked expansion along with displaced, but not resorbed, teeth, which has been reported in the literature previously [8]. This may be a helpful feature when trying to differentiate these lesions from common odontogenic tumours, such as ameloblastoma, which often show resorption of teeth.

OOCs commonly present in association with an unerupted tooth [3, 7] and this is reflected in the cases presented here, with four of the five cysts associated with an unerupted tooth. The frequency with which OOCs occur with unerupted teeth has led some authors to speculate that an OOC actually represent a dentigerous cyst with orthokeratinization, arising from reduced enamel epithelium [9]. Whilst origin from dental lamina remnants is the most likely aetiology, its pathogenesis is still uncertain [1].

Attempts have been made to explore the pathogenesis of OKC and OOCs using immunohistochemistry, with a number of studies reporting lower Ki67 expression, indicating a lower proliferation rate, in OOCs in comparison to OKCs [10–12]. It has also been shown that p63 and cyclin D1 expression is higher in OKCs [10, 11], consistent with a higher proliferation rate. Immunohistochemistry for cytokeratins shows differences between OKCs and OOCs [9, 12, 13], however the usefulness of this in clinical practice is uncertain as the distinction between an OOC and an OKC is relatively straight-forward on routine histology. However, as seen in case 1, inflammation may obscure the histology and careful analysis of the non-inflamed areas is required to arrive at a diagnosis.

Unlike OKCs, there is no evidence that multiple OOCs are associated with NBCCS [1, 14]. There is one case report of a combined OOC and OKC in a patient with Gardner Syndrome [15] and one of the extraintestinal manifestations of Gardner Syndrome is epidermoid cysts [15], which histologically can appear similar to OOCs. At the present time however, there is no convincing evidence to link multiple OOCs to NBCCS or Gardner Syndrome.

Table 1 summarises four previous reports of bilateral or multiple OOCs reported in the literature [14, 16–18]. Similar to the cases reported here, the patients were of a relatively young age, with three of the four reports involving an individual in their third decade. The majority of cases were in the mandible, with only one case reporting bilateral lesions in both the maxilla and mandible [14]. There is a male predominance and the majority of cases were associated with unerupted teeth, which is in agreement with our findings and previous studies [7]. All the cases were treated conservatively and two reported no recurrences [14, 17].

**Table 1:** Previous case reports of multiple orthokeratinized odontogenic cysts

	Location	Age	Gender	Number of cysts	Treatment	Associated with unerupted teeth	Recurrence
Pimpalkar et al. [16]	Bilateral mandible	23	Male	2	Enucleation	Yes	Not known
Cheng et al. [14]	Bilateral mandible and maxilla	23	Male	4	Three underwent curettage, one was marsupialized followed by curettage	Yes	No (14 months)
Pereira et al. [17]	Bilateral mandible	23	Female	2	Enucleation	Yes	No (27 months)
Premalatha et al. [18]	Bilateral mandible	35	Male	2	Right: enucleated. Left: decompression	No	Not known

In summary, the two unusual presentations of OOCs outlined in this paper highlight the possibility of OOCs occurring bilaterally in either the mandible or maxilla. As OOC is a relatively new entity, raising the awareness of these lesions and their ability to mimic bilateral dentigerous cysts is of importance. Further work is required to elucidate a possible link to other associated lesions in certain patients.
